# A Shift Pattern of Bacterial Communities Across the Life Stages of the Citrus Red Mite, *Panonychus citri*

**DOI:** 10.3389/fmicb.2020.01620

**Published:** 2020-07-10

**Authors:** Zhen-yu Zhang, Muhammad Waqar Ali, Hafiz Sohaib Ahmed Saqib, Sheng-xuan Liu, Xin Yang, Qin Li, Hongyu Zhang

**Affiliations:** ^1^State Key Laboratory of Agricultural Microbiology, Key Laboratory of Horticultural Plant Biology (MOE), Institute of Urban and Horticultural Entomology, College of Plant Science and Technology, Huazhong Agricultural University, Wuhan, China; ^2^Institute of Fruit and Tea, Hubei Academy of Agricultural Sciences, Wuhan, China; ^3^State Key Laboratory of Ecological Pest Control for Fujian and Taiwan Crops, Fujian Agriculture and Forestry University, Fuzhou, China; ^4^College of Life Science, Yangtze University, Jingzhou, China

**Keywords:** *Panonychus citri* (McGregor), spider mites, developmental stages, 16S rDNA amplicon pyrosequencing, dynamics of bacterial community, bacterial metabolism

## Abstract

As one of the most detrimental citrus pests worldwide, the citrus red mite, *Panonychus citri* (McGregor), shows extraordinary fecundity, polyphagia, and acaricide resistance, which may be influenced by microbes as other arthropod pests. However, the community structure and physiological function of microbes in *P. citri* are still largely unknown. Here, the high-throughput sequencing of 16S rDNA amplicons was employed to identify and compare the profile of bacterial communities across the larva, protonymph, deutonymph, and adult stages of *P. citri*. We observed a dominance of phylums Proteobacteria and Firmicutes, and classes α-, γ-, β-Proteobacteria and Bacilli in the bacterial communities across the host lifespan. Based on the dynamic analysis of the bacterial community structure, a significant shift pattern between the immature (larva, protonymph, and deutonymph) and adult stages was observed. Accordingly, among the major families (and corresponding genera), although the relative abundances of Pseudomonadaceae (*Pseudomonas*), Moraxellaceae (*Acinetobacter*), and Sphingobacteriaceae (*Sphingobacterium*) were consistent in larva to deutonymph stages, they were significantly increased to 30.18 ± 8.76% (30.16 ± 8.75%), 20.78 ± 10.86% (18.80 ± 10.84%), and 11.71 ± 5.49% (11.68 ± 5.48%), respectively, in adult stage, which implied the important function of these bacteria on the adults’ physiology. Actually, the functional prediction of bacterial communities and Spearman correlation analysis further confirm that these bacteria had positively correlations with the pathway of “lipid metabolism” (including eight sublevel pathways) and “metabolism of cofactors and vitamins” (including five sublevel pathways), which all only increased in adult stages. In addition, the bacterial communities were eliminated by using broad-spectrum antibiotics, streptomycin, which significantly suppressed the survival and oviposition of *P. citri*. Overall, we not only confirmed the physiological effects of bacteria community on the vitality and fecundity of adult hosts, but also revealed the shift pattern of bacterial community structures across the life stages and demonstrated the co-enhancements of specific bacterial groups and bacterial functions in nutritional metabolism in *P. citri*. This study sheds light on basic information about the mutualism between spider mites and bacteria, which may be useful in shaping the next generation of control strategies for spider mite pests, especially *P. citri*.

## Introduction

Many arthropods harbor diverse microbial communities in their digestive systems or intracellular/intercellular niches for symbiotic systems (Dillon and Dillon, 2004). Many of these microbiota play important roles in interactions with their hosts for improved physiology, life history traits, environmental adaptability, reproduction, and are even essential for the host’s survival (Engel and Moran, 2013; Kwong and Moran, 2016). In addition to the beneficial effect on host nutritional digestion of varied diets, especially the diets with poor or unbalanced nutrition or recalcitrant components (Hongoh et al., 2008; Huang et al., 2010), microbiota may (1) increase arthropod host fitness through protecting the host from parasites and pathogens or improving the host’s tolerance to heat stress (Dillon and Dillon, 2004; Hartman et al., 2020), (2) influence the host’s lifespan (Storelli et al., 2011; Noman et al., 2020), (3) improve the insect’s social communication (Dillon et al., 2002), and (4) govern mating and reproductive systems (Sharon et al., 2010). In some cases, microbiota facilitated the pest host’s survival under traditional pest control by enhancing pesticide resistance (Kikuchi et al., 2012; Zhou and Yao, 2020). The microbiota are even involved in the pest status of the stinkbug species, *Megacopta punctatissima* (Hosokawa et al., 2007). The diversity and structure of microbial communities in some important pests have been identified (Engel and Moran, 2013). Based on the advantage of microbial relationships with host, some new pest management strategies have been developed. For instance, commensal bacteria were genetically modified as novel biocides by expressing toxic substances, such as *Bacillus thuringiensis* toxin protein (Cyt1A) and dsRNA of critical genes for pest population persistence or expansion (Kuzina et al., 2002; Whitten et al., 2016). In some insect vectors of specific diseases (e.g., mosquito borne disease), *Wolbachia* have been successfully used in the biological control of these diseases (Pan et al., 2012).

Despite the recent massive increase in studies of the microorganisms living in arthropod guts, the mechanism of microbial biofunction is just beginning to emerge. In addition, the arthropods are the most diverse and abundant animal clade, so the diversity and function of microbial communities in the arthropods should be extremely diverse, and need more investigation in more species groups, especially in pests. The spider mites, including *Tetranychus urticae*, are one of serious agricultural pest groups in agro-ecosystems, especially for horticultural crops (Hoy and Jeyaprakash, 2005). The life cycle of spider mites consists of five stages, the egg, larva (with three pairs of legs), protonymph, deutonymph, and adult stages (with four pairs of legs in the last three stages) (Kasap, 2009), which is quite different from that of other arthropods, such as insects. Although the distributions, abundances, and bio-functions of some important genera or species of microorganisms, including bacteria, rickettsiae, fungi, and viruses, have been investigated in several spider mite species (mainly in *Tetranychus* species) (Poinar and Poinar, 1998; Hoy and Jeyaprakash, 2005; Niu et al., 2019; Ribeiro et al., 2020), only a few reports have mentioned the biofunctions and profiles of microbial communities across the life stages in spider mites (Zhu et al., 2019b), thus their biofunctions and profiles are still largely unknown. In addition, similar with other arthropods (Meng et al., 2019; Yao et al., 2019), the compositions and structures of the bacterial communities may alter across the lifespan of spider mites to adapt to the nutritional and/or physiological requirements of their hosts’ developments, which deserves investigations.

The citrus red mite, *Panonychus citri* (McGregor) (Acari: Tetranychidae), is a spider mite with worldwide distribution and is regarded as one of the most important citrus pests in many countries (Takafuji and Fujimoto, 1986). Consistent with other spider mites, it performs a lifespan consisting of egg, larva, deutonymph, protonymph and adult stages, and feeds on leaves, fruits and/or green twigs by piercing-sucking across the immature and adult stages (Zanardi et al., 2015). *P. citri* possess several critical characteristics for its environmental adaptation and plant damage, including (1) strong fertility (Ali et al., 2017), (2) a wide range of host plants (111 species of citrus and wood plants) (Gerson, 2003), and (3) high levels of resistance to various acaricides (Ran et al., 2009; Pan et al., 2020). All these characteristics facilitate this important fruit pest to live on diverse host plants with high fecundity, and also survive fatal acaricides, which may be associated with the inner-bacterial community. However, it is still unidentified the role of inner-bacterial community in the physiology and vitality of *P. citri*. Although very few researches on the symbiont bacteria have been reported, which indicated no existence of *Wolbachia* and *Cardinium* in the tested *P. citri* population (Chen et al., 2009), the structure and diversity of the microbial community across the lifespan of *P. citri* remain largely unknown. As understanding the characteristics of microbial community is the first critical step for developing the novel symbiont-based strategy of controlling pests (Mereghetti et al., 2017), the physiological function, profile and dynamics of microbial community in citrus red mite deserve investigations.

In the current study, the bacterial community structures across immature (including larva, protonymph, and deutonymph) and adult stages of citrus red mite were determined and compared by pyrosequencing of the 16S rDNA V3–V4 region, which is the next-generation DNA sequencing approach with low-cost, high throughput, and high accuracy developed in recent years (Meng et al., 2019; Yao et al., 2019). Our results indicated a significant shift pattern of bacterial communities between the immatures and adults, which may influence the predicted Kyoto Encyclopedia of Genes and Genomes (KEGG) pathways of “lipid metabolism” and “metabolism of cofactors and vitamins.” Finally, the effects of bacteria on survival and oviposition were confirmed by treatment with broad-antibiotic, streptomycin.

## Materials and Methods

### Cultivation of *P. citri*

To control the significant variation of microbiota associated with the outdoor environment, *P. citri* were collected from the sweet orange orchard (N 30° 28′ 26′′, E 114° 21′ 5′′), Huazhong Agricultural University, Hubei Wuhan, China, and reared for at least 15 populations in indoor condition as follow: the citrus red mites were kept on fresh leaves of *Citrus maxima* (Burm.) Merr., which were placed ventral-side up and surrounded by wet cotton sliver, and placed on 5 mm layer of distilled water-saturated sponge; leaf disks were renewed weekly; the temperature and moisture were controlled at 26 ± 1°C and 60 ± 5% Relative Humidity (RH), respectively and the photoperiod was 14 h (light):10 h (dark).

### *P. citri* Sample Preparation

Samples of *P. citri* at four stages, including larvae, protonymphs, deutonymphs and female adults, were collected separately in three repetitions (each repetition containing at least 300 mites). All these mites were collected from the *P. citri* cultivation originating from eggs laid in the same day (called one-day cultivations) in the same generation reared in parallel. To remove surface contaminants, each repetition of pooled mite individuals was surface-sterilized with 75% ethanol for 3 min and rinsed three times in sterile water. Then, the pool of mite individuals was used for subsequent DNA extraction for high throughput sequencing.

### DNA Extraction

The total DNA for the high-throughput sequencing was extracted by Fast DNA SPIN extraction kit (MP Biomedicals, Santa Ana, CA, United States) according to the manufacturer’s instruction, and stored at −20°C. The quality and quantity of DNA sample was determined by the agarose gel electrophoresis and NanoDrop ND-1000 spectrophotometer (Thermo Fisher Scientific, Waltham, MA, United States), respectively.

### 16S rDNA Amplicon Pyrosequencing

The V3–V4 region in bacterial 16S rDNA was amplified by PCR with the primers of 338F (5′-ACTCCTACGGGAGGCA GCA-3′) and 806R (5′-GGACTACHVGGGTWTCTAAT-3′) (Xu et al., 2016). The PCR system consisted of Q5 reaction buffer (5×, 5 μL), Q5 high-fidelity GC buffer (5×, 5 μL), Q5 high-fidelity DNA polymerase (5 U/μL, 0.25 μL), dNTPs (2.5 mM, 2 μL), each primer (10 μM, 1 μL), a total DNA template (2 μL), and ddH_2_O (8.75 μL). The PCR reaction was performed as initial denaturation (98°C for 2 min), 25 reaction cycles (each at 98°C for 15 s, 55°C for 30 s, and 72°C for 30 s), and a final extension (72°C for 5 min). PCR products were purified and quantified using Agencourt AMPure Beads (Beckman Coulter, Indianapolis, IN, United States) and PicoGreen dsDNA Assay Kit (Invitrogen, Carlsbad, CA, United States), respectively. Then, PCR amplicons were sequenced (paired-end 2 × 300 bp) using the Illlumina MiSeq platform with MiSeq Reagent Kit v3 at Shanghai Personal Biotechnology Co., Ltd. (Shanghai, China).

### Sequence Analysis

The Quantitative Insights Into Microbial Ecology (QIIME, v1.8.0) pipeline were employed to analyze the raw sequencing data as previously described (Caporaso et al., 2010). Briefly, the low-quality sequences that had average Phred scores < 20 bp, had length < 150 bp, contained ambiguous bases, and contained mononucleotide repeats > 8 bp were first filtered. Then the remained paired-end reads were assembled using Fast Length Adjustment of SHort Reads (FLASH) (Magoc and Salzberg, 2011). After chimera detection, the high-quality sequences were clustered into operational taxonomic units (OTUs) at 97% sequence identity by clustering algorithm UCLUST (Edgar, 2010), which followed by selecting a representative sequence for each OTU using default parameters. To classify the OTU taxonomy, the OTU sequences were aligned by Basic Local Alignment Search Tool (BLAST) to search the representative sequences set against the Greengenes Database using the best hit (DeSantis et al., 2006). The extremely rare OTUs (<0.001% of total sequences across all samples) were discarded. Finally, an averaged, rounded rarefied OTU table was constructed by averaging 100 evenly resampled OTU subsets under the 90% of the minimum sequencing depth to minimize the differences in sequencing depths across all samples.

### Bioinformatics Analysis

The bioinformatics analysis of sequence data were mainly performed using QIIME and R packages (v3.2.0). Alpha diversity indices (OUT level), including the Shannon diversity index, Simpson index, Berger-Parker dominance index, observed richness (Sobs), Abundance-based Coverage Estimator (ACE) metric, and Chao richness estimator, were calculated using the OTU table in QIIME. To investigate the structural variation of microbial communities across the life stage samples, beta diversity analysis was performed using weighted UniFrac distance metrics (Lozupone and Knight, 2005) and then visualized via the heatmap at class level, unweighted pair-group method with arithmetic means (UPGMA) hierarchical clustering at genus level, and principal coordinate analysis (PCoA) at OTU level (Ramette, 2007). Analysis of similarities (ANOSIM) using R package “vegan” was employed to assess the significance of differentiation of microbiota structure among groups. Based on the occurrence of OTUs across samples regardless of their relative abundance, R package “VennDiagram” was employed to generate Venn diagram for visualizing the shared and unique OTUs among different life stages. The functions of bacterial community were predicted by PICRUSt (Langille et al., 2013).

### Antibiotic Treatment via Feeding Assays

To identify the physiological function of bacterial communities on *P. citri* performance, the bacteria were eliminated by streptomycin. For antibiotic treatment, we followed a protocol that was previously established for spider mites with minor modification (Zhu et al., 2019a): selected fresh leaves were prewashed with double-distilled water and cut into small pieces of equal sizes (3 cm in diameter), then dried at a constant temperature of 45°C for 15 min. This was followed by dipping the leaf pieces in the antibiotic solutions (3 mg/ml of penicillin or streptomycin) for 1.5 h. After being dried for 3 min with a laminar flow of air, the antibiotic-treated leaves were surrounded by a wet cotton sliver and kept on sponges saturated with 1% of the antibiotic solutions. Thirty days young female adults (1–2 days after last molting), which had been starved for 3 h before use, were placed on antibiotic-treated leaves. The antibiotic-treated leaves and the antibiotic solutions for saturating sponges were changed every 3 days. All experiments were repeated three times. At each of 1, 3, 5, 7, and 9 days after the beginning of antibiotic treatment, the mite survival status (the numbers of survived and dead mites) and the number of laid eggs between the measured and the previous time points were detected. The number of laid eggs per female at each time point was calculated as (the number of laid eggs between the measured and the previous time points)/(survived female number at the measured time point)/duration. The cumulative number of laid eggs per female in 9 days was calculated by accumulating all of (the number of laid eggs per female at each time point) × duration. In addition, the total contents of nitrogen, phosphorus, potassium, and soluble sugar were determined as described in Supplementary Methods.

### Real-Time Quantitative PCR for Bacterial Counting

The mite samples (three repetitions for each time point; about 50 individuals per repetition) with or without antibiotic treatments were first surface-sterilized with 75% ethanol for 3 min and rinsed three times in sterile water. Then, the total DNA containing both bacterial genomic DNA and host DNA was extracted as mentioned above for subsequent real-time quantitative PCR.

The amounts of total bacteria were quantified by real-time quantitative PCR of partial 16S rDNA with the universal primers (Eub338F, 5′-ACTCCTACGGGAGGCAGCAG-3′; Eub518R, 5′-ATTACCGCGGCTGCTGG-3′) (Yao et al., 2019) and normalized by real-time quantitative PCR of alpha tubulin as internal control with the specific primers (TUBA-F, 5′-CGAA TCCATTTCCCCTTAGT-3′; TUBA-R, 5′-CAACGTCTCCTCG GTAAAGA-3′) (Niu et al., 2012). Each PCR mixture consisted of 10 μL of SYBR Green Mix (Bio-Rad, Hercules, CA, United States), 100 nM of each primer and 2 μL of total DNA. The amplification program consisted of preincubation at 95°C for 2 min, and 40 cycles at 95°C for 5 s and annealing at 60°C for 30 s.

### Statistical Analysis

The variations among the different stages of *P. citri* were statistically analyzed by one-way analysis of variance (ANOVA), which was followed by the *post hoc* Duncan’s Multiple Range Test (DMRT). The difference in cumulative mortality, daily or cumulative laid egg numbers between the adults with and without antibiotic-treatment was statistically analyzed by Student’s t-test. The significant correlations between bacterial species and KEGG pathways were analyzed by Spearman’s correlations and illuminated by heatmaps by GraphPad Prism 8.0 (GraphPad Software, La Jolla, CA, United States). *P*-value < 0.05 was representative of statistical significance. Results are presented as the means ± standard error of mean (SEM).

## Results

### 16S rDNA -Sequencing Data of Bacterial Communities Across the Life Stages of *P. citri*

The bacterial composition in the larvae, protonymphs, deutonymphs, and adults of *P. citri* were quantified by Illumina MiSeq platform of 16S rDNA gene amplicons. The sequencing data yielded 588,357 high-quality pyrosequencing reads with an average read length of 452.7 bp of the 16S rDNA spanning the variable regions V3–V4 from mite samples, each of which possessed 36,023–47,660 valid sequences with high quality (Table 1). Based on the 97% similarity threshold, these sequences yielded 357–724 OTUs of each sample with over 99% coverage, which indicated that most bacterial taxa (>99%) in each sample was covered (Table 1). According to the rank-abundance curves (Supplementary Figure S1A), most of the OTUs in the bacterial communities belonged to rare species. The rarefaction curves showed that the accumulation of OTUs tended toward saturation in the numbers of reads, which indicated that the samples were sufficient to reveal the bacterial communities (Supplementary Figure S1B). These results indicated that our sequencing captured most of the bacterial diversity associated with *P. citri*.

**TABLE 1:** Summary of the 16S rRNA read counts of endo-bacteria across the life stages of P. citri*.

**Sample ID**	**Read NO.**	**Mean length (bp)**	**Phylum**	**Class**	**Order**	**Family**	**Genus**	**Species**	**OUT**	**Coverage**
**L4**	48717	452.9	24	53	81	152	245	275	724	0.996
**L5**	49976	450.8	21	39	63	126	199	219	574	0.997
**L7**	54413	449.7	24	49	76	142	222	244	619	0.997
**PN4**	49231	453.3	23	51	82	152	242	267	572	0.997
**PN5**	49068	453.2	22	53	85	160	243	274	589	0.997
**PN6**	46153	445.4	22	53	79	150	226	252	497	0.997
**DN4**	52141	450.3	21	43	73	138	218	247	631	0.998
**DN5**	51455	450.3	20	42	63	120	189	214	357	0.999
**DN7**	49628	452	23	48	73	135	210	240	445	0.999
**A4**	39288	463.4	20	42	72	134	204	228	452	0.997
**A6**	43734	459.3	21	45	78	144	227	252	560	0.999
**A7**	54553	454.3	18	41	64	120	173	194	432	0.997

*L, PN, DN, and A indicated the larvae, protonymphs, deutonymphs, and adults, respectively.

### Varied Diversities of Bacterial Communities Across the Life Stages of *P. citri*

**FIGURE 1:**
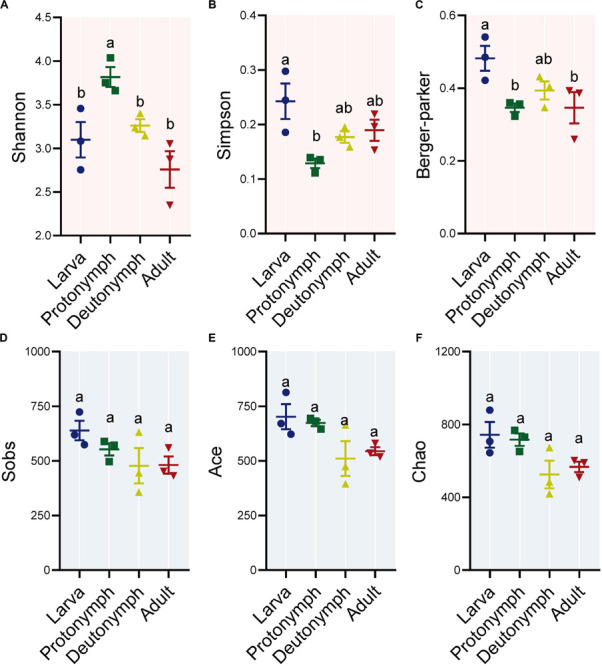
Alpha diversity of bacterial communities across the lifespan of *P. citri*. The Shannon **(A)**, Simpson **(B)**, and Berger-Parker **(C)** indexes for bacterial diversity, and observed species (Sobs, **D**), ACE **(E)** and, Chao **(F)** indexes for species richness were applied. All *n* = 3. Different lowercase letters denote significant differences between different life stages (*P* < 0.05, *post hoc* Duncan’s Multiple Range Test [DMRT]). Plots showed means ± standard error of mean (SEM).

To identify the diversity of bacteria in the larvae, protonymphs, deutonymphs, and adults of *P. citri*, six indexes were detected, including Shannon, Simpson and Berger-Parker for bacterial diversity, and observed species (Sobs), ACE and Chao for species richness. The diversity indexes were highest in larvae (Simpson and Berger-Parker) or protonymphs (Shannon), then decreased in the following life stages, especially in the adult stage with significant differences for Shannon and Berger-Parker indexes (all *P* < 0.05, *post hoc* DMRT) (Figures 1A–C). When considering the species richness indexes (i.e., Sobs, ACE, and Chao1), there was no significant difference between any stage (Figures 1D–F).

### Varied Compositions of the Bacterial Communities Across the Life Stages of *P. citri*

A Venn diagram analysis showed that subsets of 342 bacterial OTUs were shared across the lifespan of *P. citri* (Supplementary Figure S2A). According to the OTU classification based on the Greengenes Database, 24 bacterial phyla were detected in the bacterial communities of larvae, protonymphs, deutonymphs, and adults. Proteobacteria was the dominant and most diverse phylum in all samples (69.48 ± 1.57% of average relative abundance), followed by Firmicutes (11.76 ± 1.20%), Actinobacteria (7.42 ± 1.14%), Bacteroidetes (5.93 ± 1.65%), and Cyanobacteria (1.90 ± 0.53%) (Supplementary Figure S2B; see detailed phylum lists in Supplementary Table S2). Accordingly, at the class taxa level, the major classes were α-, γ-, and β-Proteobacteria (45.22 ± 4.36%, 16.96 ± 5.18%, and 7.00 ± 0.65% of relative abundances, respectively) in phylum Proteobacteria, Bacilli and Clostridia (7.09 ± 0.98% and 4.24 ± 0.44%, respectively) in Firmicutes, Actinobacteria (6.99 ± 1.12%) in Actinobacteria, and Sphingobacteria and Chitinophagia (2.96 ± 1.92% and 1.51 ± 0.36%, respectively) in Bacteroidetes (Supplementary Figure S2C; see detailed class lists in Supplementary Table S3).

**FIGURE 2:**
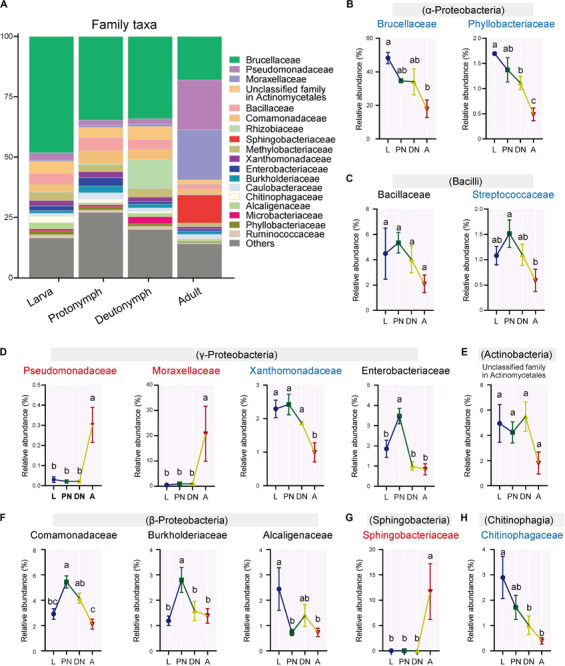
Variations of the relative abundances of bacterial families across the lifespan of *P. citri*. **(A)** Whole profiles of the relative abundances of the families in each life stage; only taxa with a relative abundance > 1% in at least one sample were analyzed. **(B–H)** Comparisons of the relative abundances of 14 major families across the larva (L), protonymph (PN), deutonymph (DN), and adult (A) stages; all *n* = 3; different lowercase letters denote significant differences between different life stages (*P* < 0.05, *post hoc* DMRT); the family names in red or blue color indicated the relative abundances of corresponding families significantly increased or decreased in the adult stage, respectively; the corresponding classes were presented in parentheses; plots showed means ± SEM.

**FIGURE 3:**
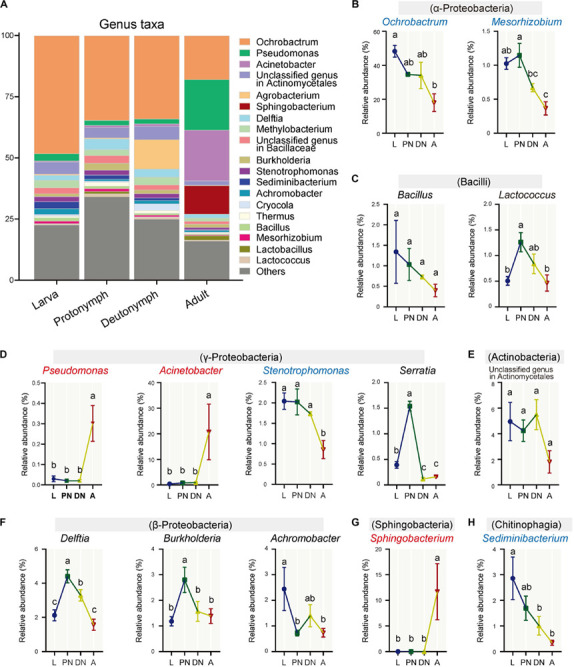
Variations of the relative abundances of bacterial genera across the lifespan of *P. citri*. **(A)** Whole profiles of the relative abundances of the genera in each life stage; only taxa with a relative abundance > 1% in at least one sample were analyzed. **(B–H)** Comparisons of the relative abundances of 14 major genera across the larva (L), protonymph (PN), deutonymph (DN), and adult (A) stages; all *n* = 3; different lowercase letters denote significant differences between different life stages (*P* < 0.05, *post hoc* DMRT); the genus names in red or blue color indicated the relative abundances of corresponding genera significantly increased or decreased in the adult stage, respectively; the corresponding classes were presented in parentheses; plots showed means ± SEM.

Furthermore, the relative abundances of the major families (and corresponding genera) varied across the lifespan of *P. citri* in six patterns (Figures 2, 3; see detailed family and genus lists in Supplementary Tables S3, S4, respectively): (1) the relative abundances of families Pseudomonadaceae (genus *Pseudomonas*), Moraxellaceae (*Acinetobacter*), and Sphingobacteriaceae (*Sphingobacterium*) were consistent in larvae, protonymphs and deutonymphs, but only significantly increased to 30.18 ± 8.76% (30.16 ± 8.75%), 20.78 ± 10.86% (18.80 ± 10.84%), and 11.71 ± 5.49% (11.68 ± 5.48%), respectively, in adults in comparison to all three immature stages; (2) the relative abundances of Brucellaceae (*Ochrobactrum*), Xanthomonadaceae (*Stenotrophomonas*), and Streptococcaceae were consistent in three immature stages, but only significantly decreased to 17.96 ± 5.21% (17.95 ± 5.19%), 1.00 ± 0.29% (0.86 ± 0.22%), and 0.59 ± 0.22%, respectively, in adults in comparison to the highest level in one or three immature stage(s); (3) the relative abundances of Phyllobacteriaceae (*Mesorhizobium*) and Chitinophagaceae (*Sediminibacterium*) were consistent in larvae and protonymphs, but significantly decreased in both deutonymphs and adults in comparison to the highest level in the larva or protonymph stage; (4) in comparison to that of larvae, the relative abundances of Enterobacteriaceae (*Serratia*), Comamonadaceae (*Delftia*), Burkholderiaceae (*Burkholderia*), and genus *Lactococcus* were all significantly increased in protonymphs, then dropped in deutonymph and adult stages to the levels similar to that of larvae stage, except for significantly lower levels of *Serratia* in deutonymph and adult stages than that of larva stage; (5) the relative abundances of Alcaligenaceae (*Achromobacter*) were highest in larvae, and significantly decreased in the followed three stages; (6) the relative abundances of Bacillaceae (*Bacillus*), unclassified family in Actinomycetales (unclassified genus in Actinomycetales), and other major classes (genera) were consistent across the life stages (*P* < 0.05 for all significant differences, *post hoc* DMRT).

### Varied Structures of the Bacterial Communities Across the Life Stages of *P. citri*

**FIGURE 4:**
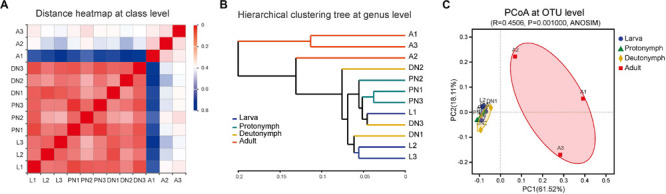
Bacterial community dissimilarity across the lifespan of *P. citri* based on weighted UniFrac metrics. **(A)** Sample distance heatmap analysis at class level. **(B)** Analysis of unweighted pair-group method with arithmetic mean (UPGMA) based on hierarchical clustering at genus level. **(C)** Principal coordinated (PCoA) analysis at OTU level; the significance of variation in communities across four life stages was analyzed by analysis of similarities (ANOSIM) test with 999 permutations. L, PN, DN, and A indicated the larva, protonymph, deutonymph, and adult stages, respectively.

Based on the compositions of the bacterial communities across the life stages, the community dissimilarities in class, genus, and OTU taxa levels were investigated further. According to weighted UniFrac metrics, the heatmap analysis at the class level (Figure 4A), the analysis using the UPGMA based on hierarchical clustering at genus level (Figure 4B), and PCoA analysis at the OTU level (Figure 4C) were constructed. All analyses indicated that community structures of bacteria in the larvae, protonymphs, and deutonymphs were similar and clustered into one group, and that in the adults were different and clustered into another group (except that adult sample A2 was more similar to immature samples than the other two adult samples, according to hierarchical clustering analysis at the genus level). Moreover, the analysis of similarities (ANOSIM) results indicated a significant difference in the bacterial community of *P. citri* across the lifespan (*R* = 0.4506 and *P* = 0.001; Figure 4C), which supported the clustering of the bacterial communities over the four life stages into two groups.

### Varied Functions of the Bacterial Community Across the Life Stages of *P. citri* Based on PICRUSt Metagenomic Prediction

**FIGURE 5:**
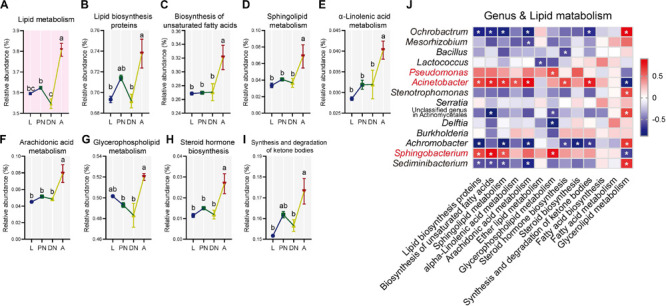
Dynamics of predicted KEGG pathways of “lipid metabolism” and sublevel pathways across the lifespan of *P. citri*. **(A–I)** The “lipid metabolism” pathway at level two (A) and eight sublevel pathways at level three **(B–I)** significantly varied across the larva (L), protonymph (PN), deutonymph (DN), and adult (A) stages; all *n* = 3; different lowercase letters denote significant differences between different life stages (*P* < 0.05, *post hoc* DMRT); plots showed means ± SEM. **(J)** Heatmaps based on Spearman’s correlation analysis between the relative abundances of “lipid metabolism” pathways and major genera; the red and blue represent positive and negative correlations between the relative abundances of pathways and bacterial genera, respectively; *indicated *P* < 0.05, Spearman’s correlation.

**FIGURE 6:**
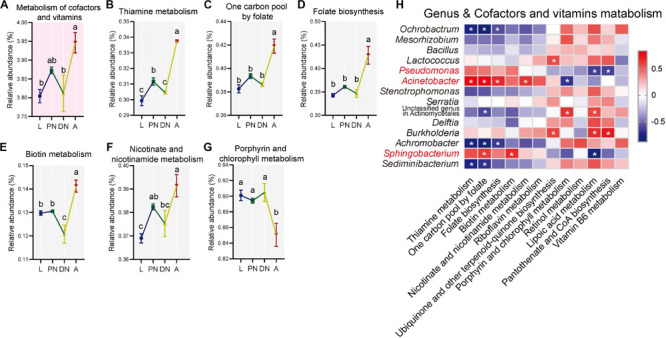
Dynamics of predicted KEGG pathways of “metabolism of cofactors and vitamins” and sublevel pathways across the lifespan of *P. citri*. **(A–G)** The “metabolism of cofactors and vitamins” pathway at level two (A) and five sublevel pathways at level three **(B–G)** significantly varied across the larva (L), protonymph (PN), deutonymph (DN), and adult (A) stages; all *n* = 3; different lowercase letters denote significant differences between different life stages (*P* < 0.05, *post hoc* DMRT); plots showed means ± SEM. **(H)** Heatmaps based on Spearman’s correlation analysis between the relative abundances of “metabolism of cofactors and vitamins” pathways and major genera; the red and blue represent positive and negative correlations between the relative abundances of pathways and bacterial genera, respectively; *indicated *P* < 0.05, Student’s *t*-test.

Using PICRUSt metagenomic prediction, 7, 37, and 290 KEGG pathways were obtained at level 1, 2, and 3, respectively (see detailed lists in Supplementary Tables S5–S7, respectively). Among the major KEGG pathways at level 2 (relative abundance > 1%), the relative abundances of “lipid metabolism” (Figure 5A) and “metabolism of cofactors and vitamins” (Figure 6A) were consistent within larvae, protonymphs, and deutonymphs, but significantly increased in adults in comparison to that of immatures (*P* < 0.05 for all significant differences, *post hoc* DMRT). Meanwhile, similar patterns were observed in other six pathways, including “folding, sorting and degradation,” “replication and repair,” “transcription,” “translation,” “glycan biosynthesis and metabolism,” and “metabolism of terpenoids and polyketides” (Supplementary Figure S3).

To investigate the KEGG pathways related to the physiology of host adults, the “lipid metabolism” and “metabolism of cofactors and vitamins,” which have been identified to be important for the vitality and fecundity of host (Douglas, 2015; Ali et al., 2017; Zhang X. et al., 2018; Canfora et al., 2019), were selected for further analysis. In the “lipid metabolism,” the relative abundances of eight pathways at level 3, including “lipid biosynthesis proteins,” “biosynthesis of unsaturated fatty acids,” “sphingolipid metabolism,” “α-linolenic acid metabolism,” “arachidonic acid metabolism,” “glycerophospholipid metabolism,” “steroid hormone biosynthesis,” and “synthesis and degradation of ketone bodies,” were consistent in larvae, protonymphs and deutonymphs, but significantly increased in adults; and other pathways were consistent across the lifespan (Figures 5B–I; *P* < 0.05 for all significant differences, *post hoc* DMRT). In terms of the “metabolism of cofactors and vitamins,” the relative abundances of five pathways at level 3, including “thiamine metabolism,” “one carbon pool by folate,” “folate biosynthesis,” “biotin metabolism,” and “nicotinate and nicotinamide metabolism,” were consistent in the three immature stages, but significantly increased in adults; the “porphyrin and chlorophyll metabolism” was consistent in the three immature stages, but significantly decreased in adults; and other pathways were consistent across the lifespan (Figures 6B–G; *P* < 0.05 for all significant differences, *post hoc* DMRT). The detailed information of dynamics of other six KEGG pathways mentioned above was presented in the Supplementary Table S8.

To clarify the association between the alteration of the composition and predicted functionality of the bacterial communities, Spearman’s correlation analysis was performed. The results showed that relative abundances of genera *Pseudomonas*, *Acinetobacter*, and *Sphingobacterium* had positive correlations with almost all the predicted pathways for “lipid metabolism,” which with one, seven, and four significant positive correlations, respectively. In addition, the same genera had positive correlations with half of the predicted pathways for “metabolism of cofactors and vitamins” (including all pathways performed with significant increases in the adult stage), which with zero, four, and two significant positive correlations, respectively (Figures 5J, 6H). In contrast, the relative abundances of *Ochrobactrum*, *Mesorhizobium*, *Stenotrophomonas*, *Sediminibacterium*, and *Achromobacter* had negative correlations with almost all the predicted pathways for “lipid metabolism,” which with five, one, zero, four, and six significant positive correlations, respectively. In addition, these genera had negative correlations with half of the predicted pathways for “metabolism of cofactors and vitamins” (including all pathways demonstrating with significant increases in the adult stage), which with three, zero, zero, two, and three significant negative correlations, respectively (Figures 5J, 6H).

### Abundance of Symbionts Across the Life Stages of *P. citri*

According to the symbionts reported in other spider mites (such as the *Tetranychus* species) and sap-feeding insects (such as aphids and psyllids) (Meyer and Hoy, 2008; Zhao et al., 2016; Staudacher et al., 2017; Meng et al., 2019), the sequencing reads of *Wolbachia*, *Carsonella*, *Arsenophonus*, *Profftella*, *Oxalobacter*, *Herbaspirillum*, *Rickettsia*, *Cardinium*, *Spiroplasma*, *Buchnera*, *Hamiltonella*, *Regiella*, *Serratia*, and *Spiroplasma* were screened in the sequencing data. No or almost no read for each symbiont was detected across the life stages, except that *Serratia* had very low relative abundances (0.0061–1.263%) across the life stages, which was significantly highest in protonymphs (Table 2).

**TABLE 2:** Sequencing reads of symbionts across the life stages of P. citri*.

**Species**	**Larva**	**Protonymph**	**Deutonymph**	**Adult**	**References**
*Wolbachia*	0	<0.0001	0	0	Zhao et al., 2016; Staudacher et al., 2017; Meng et al., 2019
*Carsonella*	0	0	0	0	Meyer and Hoy, 2008; Meng et al., 2019
*Arsenophonus*	0	0	0	0	Zhao et al., 2016
*Profftella*	0	0	0	0	Meyer and Hoy, 2008; Meng et al., 2019
*Oxalobacter*	0	0	0	0	Meyer and Hoy, 2008
*Herbaspirillum*	<0.0001	0	<0.0001	<0.0001	Meyer and Hoy, 2008
*Rickettsia*	0	0	0	0	Zhao et al., 2016; Staudacher et al., 2017
*Cardinium*	0	0	0	0	Staudacher et al., 2017
*Spiroplasma*	0	0	0	0	Staudacher et al., 2017
*Buchnera*	0	0	0	0	Zhao et al., 2016
*Hamiltonella*	0	0	0	0	Zhao et al., 2016
*Regiella*	0	0	0	0	Zhao et al., 2016
*Serratia*	0.00276 ± 0.00038^(b)^	0.01161 ± 0.00082^(a)^	0.00083 ± 0.00021^(c)^	0.00127 ± 0.00015^(bc)^	Zhao et al., 2016
*Spiroplasma*	0	0	0	0	Zhao et al., 2016

*L, PN, DN, and A indicated the larvae, protonymphs, deutonymphs, and adults, respectively. Different lowercase letters in parentheses denote significant differences between different life stages (*P* < 0.05, post-Duncan’s test).

### Bacterial Elimination by Antibiotic Treatment Suppress the Survival and Oviposition of *P. citri*

**FIGURE 7:**
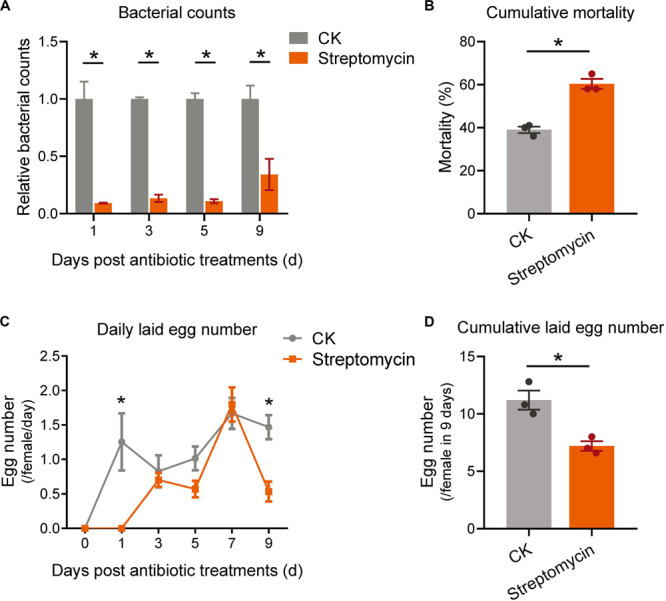
The effects of antibiotic-treatments on the survival and reproduction of *P. citri*. After treatment of streptomycin (3 mg/ml), the relative bacterial counts **(A)**, cumulative mortalities **(B)**, daily **(C)**, and cumulative laid egg number **(D)** were detected and compared to that of control (CK). All *n* = 3. The bacterial counts at each time point in (A) were normalized to the mean of CK to calculate the relative bacterial counts. *Indicated the significant difference between streptomycin treatment and CK. Plots showed means ± SEM.

Finally, to identify the physiological function of bacterial communities on adult performance of *P. citri*, the bacteria in host adults were eliminated by broad-spectrum antibiotics, streptomycin, which has been used in eliminating the bacteria in some insect pests and stored-product mites (Kopecky et al., 2014; Paramasiva et al., 2014; Lin et al., 2015). Under the antibiotic treatment, the relative bacterial counts were significantly decreased to 9.13 ± 0.38% of control (without antibiotic treatment) at 1 day, then kept at 13.37 ± 3.14%, 10.84 ± 1.75%, and 34.09 ± 13.67% at 3, 5, and 9 days, respectively (Figure 7A; all *P* < 0.05, Student’s *t*-test). In comparison to control, although the daily mortalities were not significantly different under antibiotic treatments at each time point of 1, 3, 5, 7, and 9 days (Supplementary Figure S4), the cumulative mortality at 9 days was significantly increased by 54.7% in streptomycin-treatment groups (Figure 7B; *P* < 0.05, Student’s *t*-test). In addition, the daily numbers of laid eggs were significantly reduced at 1 and 9 days (Figure 7C; all *P* < 0.05, Student’s *t*-test); therefore the cumulative number of laid eggs was significantly decreased by 35.7% at 9 days after streptomycin treatments (Figure 7D; *P* < 0.05, Student’s *t*-test). To exclude the possibility of the adverse effect of antibiotics on the nutritional condition of citrus leaves to influence the mite performance, the appearances and major nutrition contents (including the total contents of nitrogen, phosphorus, potassium, and soluble sugar) of citrus leaves were determined (see the detailed methods in Supplementary Methods), which indicated no significant differences in appearances and major nutrition contents after antibiotic-treatments (Supplementary Figure S5 and Supplementary Table S9). These results indicated that the survival and fertility of *P. citri* were reduced by eliminating bacteria, rather than by aggravating the nutritional status of citrus leaves, under broad-spectrum antibiotic treatments.

## Discussion

In contrast to insect pests, little is known about the physiological effects of the microbial community on the spider mite performance and its composition across different development stages (Poinar and Poinar, 1998; Hoy and Jeyaprakash, 2005; Ribeiro et al., 2020), especially in the detrimental pest, citrus red mites. Here, the compositions and structures of the bacterial communities were revealed by high-throughput sequencing of 16S rDNA and compared across the development stages, which indicated a significant shift pattern between the immature and adult stages of *P. citri*. Accordingly, the relative abundances of family Pseudomonadaceae (genus *Pseudomonas*), Moraxellaceae (*Acinetobacter*), and Sphingobacteriaceae (*Sphingobacterium*) were consistent in larva to deutonymph stages, but significantly increased in adult stage. Furthermore, the bacterial functions of the “lipid metabolism” and “metabolism of cofactors and vitamins” pathways were predicted to be enhanced in the adult stage, and significantly positively correlated with the coenhancements of Pseudomonadaceae (*Pseudomonas*), Moraxellaceae (*Acinetobacter*), and Sphingobacteriaceae (*Sphingobacterium*), which implies that the enhancements of these bacteria may contribute to adapt to the physiological requirement of host adults by strengthening the metabolisms of lipid, cofactors and vitamins. To preliminarily test this implication, the broad-spectrum antibiotics, streptomycin, was used to eliminate the bacterial community in adult stage of *P. citri*. The results indicated the significant effects of bacterial community on the physiology of *P. citri*, such as survival and fertility. Our results not only confirmed the physiological effects of bacteria in *P. citri*, but also elucidated the profiles of bacterial communities and the shift pattern of community structures. In addition, the abundance enhancements of several specific bacteria, including Pseudomonadaceae (genus *Pseudomonas*), Moraxellaceae (*Acinetobacter*), and Sphingobacteriaceae (*Sphingobacterium*), were correlated with the physiological requirement of host adult and predicted to contribute to the adult vitality and fecundity of *P. citri*. This facilitates our understanding of the mutualism between host and bacteria, and may contribute to shaping potential biocontrol approaches against *P. citri* and other spider mite pests.

In *P. citri*, the phylum Proteobacteria (with α- and γ-Proteobacteria as the major classes) and Firmicutes (with Bacilli and Clostridia) were dominant and diverse across the developmental stages, which is consistent with other spider mites and many insect species in Lepidoptera, Diptera, Coleoptera, Hymenoptera and Hemipter (Yun et al., 2014; Zhang Z.Y. et al., 2018; Zhao et al., 2018; Meng et al., 2019; Yao et al., 2019), but quite different from some sap-feeding insects, such as aphids (Gauthier et al., 2015; Zhao et al., 2016). Proteobacteria species are important in nitrogen fixation, metabolisms of critical nutritional components (including sugars and proteins), insecticide resistance, and protection against parasites and pathogens in fruit flies, aphids, and moths (Oliver et al., 2003; Behar et al., 2005; Xia et al., 2013; Zhao et al., 2018; Meng et al., 2019; Yao et al., 2019). Thus, the α- and γ-Proteobacteria species in *P. citri* were predicted to be important in the degradation and use of plant materials for nutritional supply and anti-pathogens for its hosts. Meanwhile, the Bacilli and Clostridia in Firmicutes may also play important roles in host development, detoxification of plant toxic compounds, and anti-pathogens, which has been identified in *Drosophila*, moths, and bees (Shin et al., 2011; Storelli et al., 2011; Xia et al., 2013; Mereghetti et al., 2017; Shao et al., 2017). These results imply that broad-spectrum antibiotic treatment may influence the vitality and fecundity of *P. citri* by eliminating the dominant bacteria, such as Proteobacteria and Firmicutes species. However, some major families and genera discovered in *P. citri* in our study rarely exist in other spider mites and insects. For instance, the most dominant family Brucellaceae (genus *Ochrobactrum*) consisted of 33.71 ± 3.87% of the average relative abundance in the bacterial community of *P. citri*. The Brucellaceae family was considered as environmental bacteria and only reported in mammals as opportunistic pathogens and gut bacterial species in dipteran *Lutzomyia longipalpis*, which is the vector of *Leishmania infantum* (the leishmaniasis pathogen) (Kelly et al., 2017; Zheng et al., 2019). These uncommon bacteria species indicate that the bacterial community in this spider mite pest is distinct from that in other spider mites or insects, and may be determined by the host’s diets and/or physiological requirements for host development and reproduction (Engel and Moran, 2013; Mereghetti et al., 2017).

Across the life stages, the bacterial diversity showed a declining trend in *P. citri* (Figures 1A–C), which is consistent with that in some insects, such as psyllids, fruit flies, planthoppers, and ladybirds (Yun et al., 2014; Meng et al., 2019; Wang et al., 2019; Yao et al., 2019). The higher bacterial diversity in the immature stages of insect has been associated with the diverse environmental microbes originating from the eggs and/or diets; and during the development, some exogenous bacteria may be unable to adjust the hosts’ digestive tracts in the adult stage. Thus, these bacteria perform as transient microbes, which may induce the decrease of bacterial diversity (Meng et al., 2019; Yao et al., 2019). This implies that some major bacterial families and genera in *P. citri*, which significantly reduced in the adult stage (Figures 2, 3), originated from eggs and/or plant diets and colonized in the internal niches (such as digestive tracts) during immature stages, but reduced in adult stage because of the declined adaptability to the internal niches in this spider mite. To clarify this phenomenon in *P. citri*, a comparison of microbe communities from all stages (including egg stage) and diets (such as the citrus leaves) deserves further investigation.

Cluster analysis showed a clear separation of the adults’ bacterial community from immatures (including larvae, protonymphs and deutonymphs) of *P. citri* (Figure 4). This shift pattern of bacterial community structures between immature and adult stages was discovered in acarid species for the first time, and is similar to many insects, including both holometabolous and hemimetabolous species (Zhao et al., 2018; Meng et al., 2019; Nobles and Jackson, 2020). In holometabolous insects, the diets and habitats are usually extremely varied across the larvae, pupa to adults, and are considered as critical factors for inducing extreme transmission between immatures and adults (Zhao et al., 2018; Yao et al., 2019). However, the diets and habitats were consistent across the life stages of *P. citri* (Zanardi et al., 2015). Therefore, other factors, such as alterations in physiology, immune system, and/or phylogeny across the developmental stages (Yun et al., 2014; Yao et al., 2016), probably play important roles in the shift pattern of bacterial community in *P. citri*, which has been identified in hemimetabolous insects, such as psyllids, planthoppers, and whiteflies (Indiragandhi et al., 2010; Meng et al., 2019; Wang et al., 2019).

Consistent with the alteration of the bacterial community structure, some major families/genera and predicted bacterial metabolic pathways including “lipid metabolism” and “metabolism of cofactors and vitamins” were co-enhanced in the adult stages in comparison to other immature stages, and performed with significant positive correlations (Figures 5, 6). These major families/genera include families Pseudomonadaceae (genus *Pseudomonas*), Moraxellaceae (*Acinetobacter*), and Sphingobacteriaceae (*Sphingobacterium*) (Figures 2, 3, 5, 6). Considering the critical role of lipid accumulation in the fecundity of *P. citri* (Ali et al., 2017), enhanced bacterial function in lipid metabolism should facilitate host lipid production, which has been supported by the significant influence of bacteria-produced short chain fatty acids on the host lipid metabolism or glucose-lipid homeostasis as previous reports (Zhang X. et al., 2018; Canfora et al., 2019). As well, the bacterial metabolisms of cofactors and vitamins are important for insect survival and reproduction (Douglas, 2015), which reveals the potential role of the bacterial metabolism of cofactors and vitamins in the vitality and fecundity of *P. citri*. Furthermore, the significantly positive correlations between the bacterial predicted functions and these families (and genera) implies that abundance enhancements of Pseudomonadaceae (*Pseudomonas*), Moraxellaceae (*Acinetobacter*), and Sphingobacteriaceae (*Sphingobacterium*) may contribute to the enhancements of bacterial “lipid metabolism” and “metabolism of cofactors and vitamins,” therefore facilitate the survival and reproduction in adult host. Consistently, the elimination of bacteria by antibiotic-treatment suppressed the vitality and fertility of *P. citri*, which preliminarily supports this implication. Previous reports have discovered that the Sphingobacteriales abundance in compost may be influenced by fatty acid content (Reddy et al., 2018), *Pseudomonas* isolates consisted of nutritionally versatile chemoorganotrophs (such as lipase) to metabolize a very wide range of organic compounds (such as lipids) *in vitro* (Gilbert, 1993; Litthauer et al., 2002), and *Acinetobacter calcoaceticus* isolate could use short chain monocarboxylic acids *in vitro* (Du Preez et al., 1985). However, it is the first study to establish the positive correlation between specific species of Proteobacteria/Sphingobacteria and the metabolisms of lipid, cofactors and vitamins, which may be critical for the survival and reproduction of spider mites and deserves further investigation. In contrast, the relative abundances of some major families and genera decreased in the adult stage (including family Brucellaceae [genus *Ochrobactrum*], Xanthomonadaceae [*Stenotrophomonas*], Streptococcaceae, Phyllobacteriaceae [*Mesorhizobium*], and Chitinophagaceae [*Sediminibacterium*]) or fluctuated across the lifespan (including Enterobacteriaceae [*Serratia*], Comamonadaceae [*Delftia*], Burkholderiaceae [*Burkholderia*], Alcaligenaceae [*Achromobacter*], and genus *Lactococcus*) (Figures 3, 4). This implies that these bacteria are less important for the survival and fertility of adult mites. These bacteria may originate from the eggs and/or native plant diet, and perform as transient microbes with specific functions at immature stages (Mereghetti et al., 2017; Yao et al., 2019).

Unexpectedly, no or almost no obligate and facultative symbiont was detected in all stages, except for *Serratia* with very low abundance across the life stages of *P. citri*. This result is consistent with previous reports on other *P. citri* populations (Gotoh et al., 2003; Chen et al., 2009), but differs from some other spider mites (such as several *Tetranychus* species) and sap-feeding insects (such as psyllids and aphids) (Gauthier et al., 2015; Zhao et al., 2016; Meng et al., 2019; Ribeiro et al., 2020). In fact, different from persistence of symbionts in sap-feeding insects which are essential for the host’s development and survival (Zhao et al., 2016; Meng et al., 2019), the symbiont is absent in some spider mites (Gotoh et al., 2003), including *P. citri* in our study, which indicates the unnecessity of these symbionts for the life history traits of these arthropod hosts. Therefore, in *P. citri*, we predicted that the specific bacteria species in Pseudomonadaceae (*Pseudomonas*), Moraxellaceae (*Acinetobacter*), and Sphingobacteriaceae (*Sphingobacterium*) identified in our study, rather than any known bacterial symbionts, play important roles in the survival and reproduction of *P. citri*, which deserves further investigation.

Our results indicated the physiological functions of bacteria community on the vitality and fecundity in *P. citri* adults by eliminating the bacteria using broad-spectrum antibiotics. Dissimilar from using tetracycline to eliminate specific symbionts (such as *Wolbachia*) in *Tetranychus* species (Staudacher et al., 2017; Zhu et al., 2019a), the current study used streptomycin to reduce the total bacterial accounts because of the absence of the symbionts which have been reported in other spider mites and sap-feeding insects (Meyer and Hoy, 2008; Zhao et al., 2016; Staudacher et al., 2017; Meng et al., 2019). Therefore, we just inferred that the abundance enhancements of Pseudomonadaceae (*Pseudomonas*), Moraxellaceae (*Acinetobacter*), and Sphingobacteriaceae (*Sphingobacterium*) may contribute to the adult vitality and fecundity of *P. citri*, which needs further clarification. In many cases, the bacteria in the insect hosts were eliminated by bactericidal antibiotic treatments to clarify the bacterial biofunction on the hosts (Lin et al., 2015; Staudacher et al., 2017), which is consistent with our study. However, we still cannot exclude the adverse effects of bactericidal antibiotics on the host physiology by inducing mitochondrial dysfunction and oxidative damage to cell, which has been observed in studies of mammals (Kalghatgi et al., 2013). To avoid the adverse influence of antibiotic treatment and confirm the bacterial physiological functions on *P. citri*, generation of germ-free mites by rearing in a sterile environment can be employed (Bing et al., 2018), which deserves further investigation.

## Conclusion

The present study not only confirmed the physiological function of bacteria on spider mite’s vitality and fertility, but also revealed the profiles of bacterial communities, which greatly differed between the immature and adult stages in *P. citri*. Additionally, the abundance enhancements of families Pseudomonadaceae (genus *Pseudomonas*), Moraxellaceae (*Acinetobacter*), and Sphingobacteriaceae (*Sphingobacterium*) in adult stage were identified and positively correlated to the enhancement of bacterial KEGG pathways of lipid, cofactor and vitamin metabolism, which implies that a novel mechanism potentially exists for commensalism of mites and bacteria to facilitate the hosts’ survival and reproduction. Reaching similar determinations using various populations collected from different habitats (including ourdoor/natural environments) and plants will facilitate in better understanding the characteristics of core microbes and the dynamics of the bacterial community that are affected by environmental factors, which are worth investigating further.

## Data Availability Statement

The datasets presented in this study can be found in online repositories. The names of the repository/repositories and accession number(s) can be found at: https://www.ncbi.nlm.nih.gov/genbank/, PRJNA608605.

## Author Contributions

ZZ contributed to the experimental design and implementation, data analysis, and manuscript preparation and submission. HZ conceived and designed the laboratory experiments. ZZ conducted the main experiments and collected, analyzed, and interpreted the data. MA, HS, SL, XY, and QL assisted in parts of the experiments. ZZ, MA, and HZ wrote the manuscript. All authors contributed to the article and approved the submitted version.

## Conflict of Interest

The authors declare that the research was conducted in the absence of any commercial or financial relationships that could be construed as a potential conflict of interest.
