# Supplementary material for: A Shift Pattern of Bacterial Communities Across the Life Stages of the Citrus Red Mite, Panonychus citri
Source: Front Microbiol. 2020 Jul 10;11:1620. doi: 10.3389/fmicb.2020.01620 (PMC7366552; doi:10.3389/fmicb.2020.01620)
Supplement: Supplementary file 2 [file Data_Sheet_1.docx]

**Supplementary Methods**

**Determination of** **Total Contents of Nitrogen, Phosphorus, Potassium in Citrus Leaves**

After washing with ddH2O twice, the citrus leaves of *Citrus maxima* (Burm.) Merr. were first dried at 80°C overnight, and then determined the total contents of nitrogen, phosphorus, potassium according to the previous methods (Ning et al., 2013).

**Determination of Total Soluble Sugar Content**

After being cutted into pecieces, the fresh leave sample was devided into two parts, which were used for both determinations of the total soluble sugar content and water content. To determine the total soluble sugar content (percentage in fresh leave mass), the shredded leaves was homogenated and centrifugated as 10000 × g at 4 for 5 mins, then the total soluble sugar content in the supernatant was detected using the Plant Suger Content Kit (Jiancheng Bio Co., Nanjing, China). To determine the water content, another part of the shredded leaves was dried at 80°C overnight, and the water content was calculated as (dried leave weight) / (fresh leave weight). Finally, the total soluble sugar content (percentage in dried leave mass) was the calculated as (percentage in fresh leave mass) / (1- water content).

**References**

Ning, P., Li, S., Yu, P., Zhang, Y., and Li, C. (2013). Post-silking accumulation and partitioning of dry matter, nitrogen, phosphorus and potassium in maize varieties differing in leaf longevity. *Field Crop. Res.* 144, 19-27. doi: https://doi.org/10.1016/j.fcr.2013.01.020.

**Figure S1**


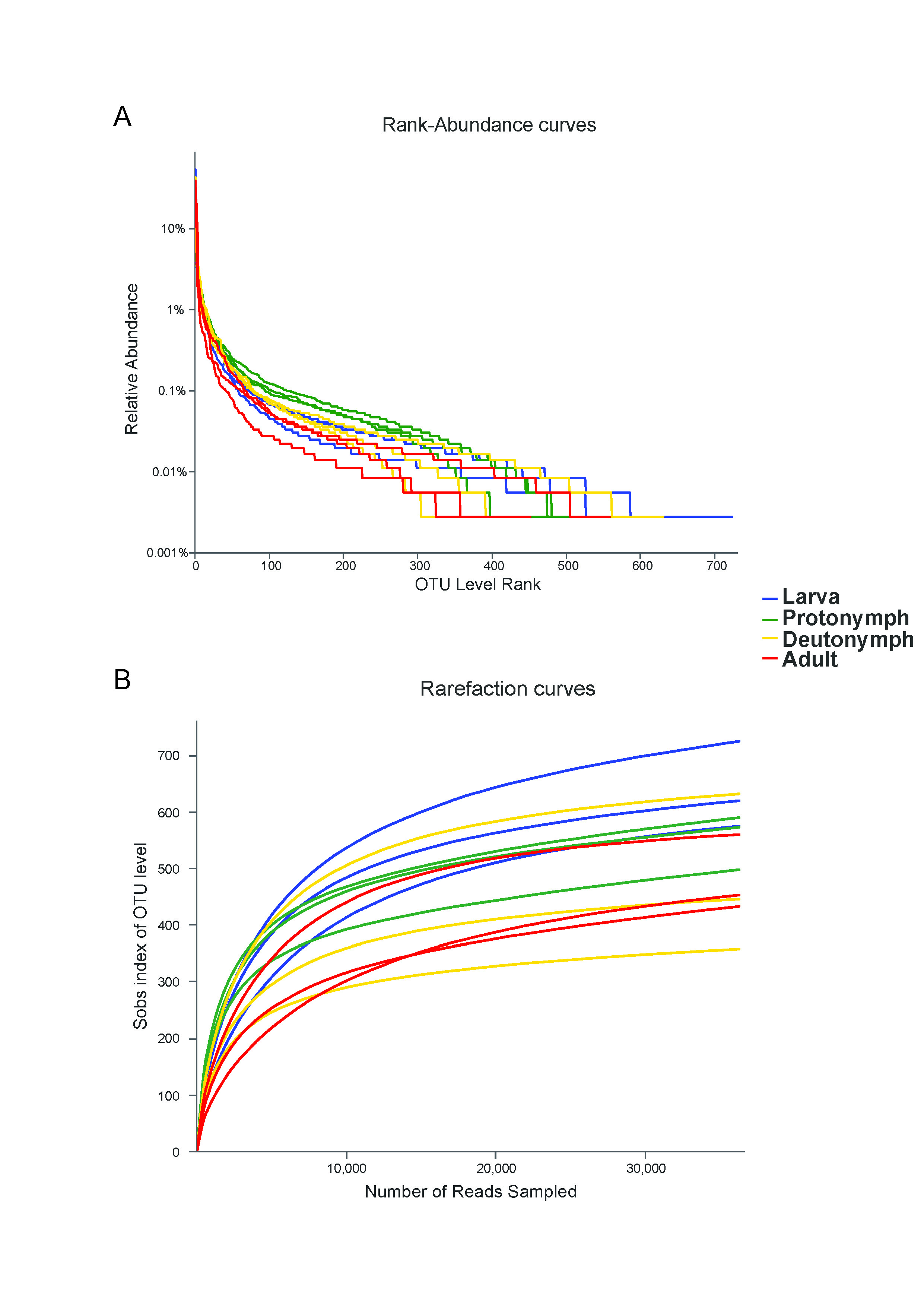


**Figure S1 Rank-abundance curves (A) and rarefaction curves (B) of bacteria based on bacterial OTUs at a similarity level of 97%**.

**Figure S2**

**Figure S2. The structures of bacterial communities across the lifespan of *P. citri*.** (A) Overlap of OTUs between different life stages. (B–C) Whole profiles of the relative abundances of the phylums (B) and classes (C) in each life stage; only taxa with a relative abundance > 0.1% in at least one sample were analyzed.

**Figure S3**

**Figure S3. Dynamics of the predicted KEGG pathways at level 2 across the lifespan of *P. citri*.** All n = 3. Different lowercase letters denote significant differences between different life stages (*P* < 0.05, post-hoc Duncan’s Multiple Range Test [DMRT]). Plots showed means ± SEM.

**Figure S4**


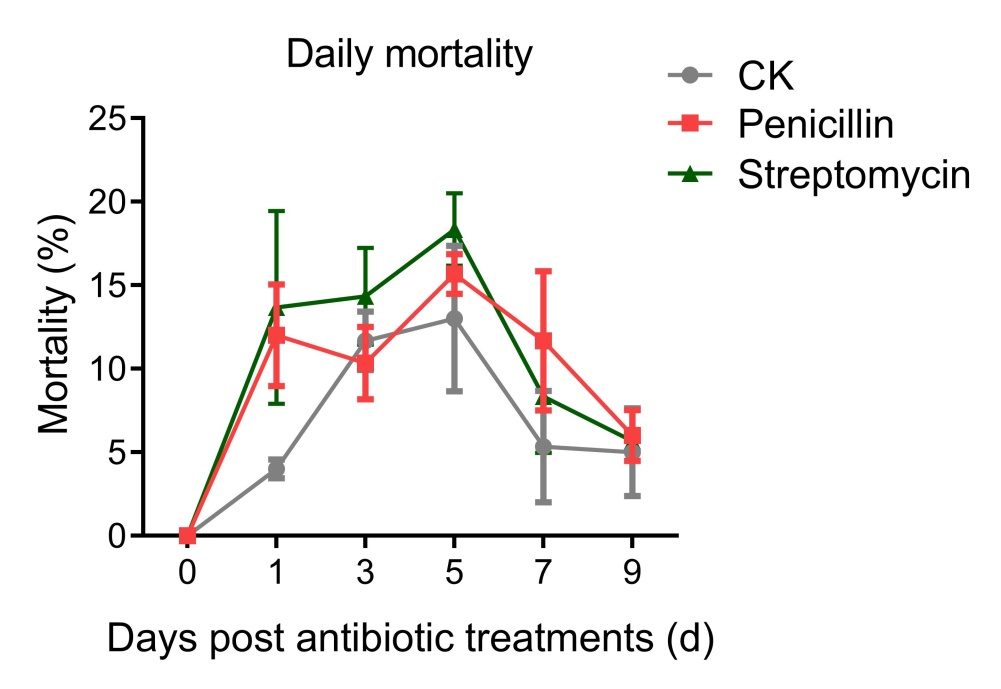


**Figure S4 The effects of antibiotic-treatments on the daily mortalities of *P. citri*.** The antibiotic-treatments were performed with penicillin (3 mg/ml) and streptomycin (3 mg/ml). All n = 3. CK indicated control. Plots showed means ± SEM..

**Figure S5**


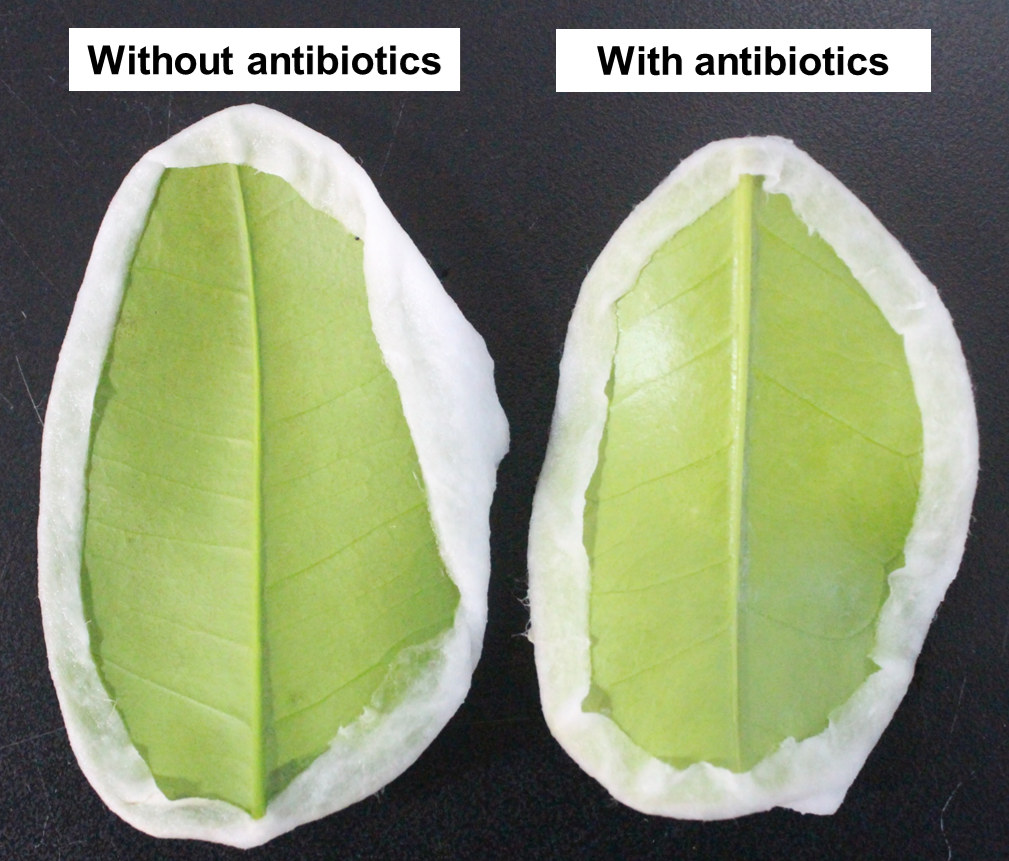


**Figure S5 Appearances of the fresh leaves of *Citrus maxima* (Burm.) Merr with or without antibiotic-treatment after keeping 3 d.**
